# The Euregional policy impact of COVID 19 on the border region between the Netherlands, North-Rhine Westphalia and Belgium

**DOI:** 10.1093/eurpub/ckac129.215

**Published:** 2022-10-25

**Authors:** C Hoebe

**Affiliations:** 1 GGD, Zuid-Limburg, Heerlen, Netherlands; 2 Department of Social Medicine, University of Maastricht, Maastricht, Netherlands

## Abstract

During the Coronavirus pandemic, internal European borders were temporarily re-established with the argument to mitigate the outbreak. Also in the border region between the Netherlands, North-Rhine Westphalia and Belgium (EMR). Existing evidence on the effectiveness of border control for infectious disease control (IDC) has been dominated by studies that focused on scenarios within countries with limited attention to border regions. To address this gap, we analysed the experiences of public health professionals working in European border regions. We conducted three studies: 1. seroprevalence and questionnaire study among 10.001 Dutch persons with and without cross border mobility, 2. analyses of incidence data in municipalities in 4 cross border regions to analyse cross border differences, and 3. we conducted 27 semi-structured interviews with public health professionals in the EMR. Participants were asked about their perspectives on border controls and the spread of Covid-19.

Four key-results: First, border regions are characterised by dynamic social life and cross-border movements. Incidence was mainly determined by country policy, Second, the impact of border control and closing on local infectious disease epidemics is likely marginal. Third, due to the dynamic social life, border control measures cannot be fully implemented in border regions, and thus their effectiveness is even more questionable. Fourth, border control measures may harm the social fabric of border regions more than they do in in-countries territories. Our study results highlighted the ineffective role of border control measures for regional infectious disease control. Sustainable cross-border collaboration is crucial to ensure effective pandemic management in border regions. The results of our study impacted on policy makers, to be much more reluctant with closing borders.

